# Effects of a Psycho‐Behavioural Intervention on Cancer‐Related Fatigue Among Patients With Colorectal Cancer: A Pilot Randomised Controlled Trial

**DOI:** 10.1002/pon.70570

**Published:** 2026-08-07

**Authors:** Jinling Lu, Yuen Yu Chong

**Affiliations:** ^1^ Faculty of Medicine The Nethersole School of Nursing The Chinese University of Hong Kong Hong Kong China

**Keywords:** cancer‐related fatigue, colorectal cancer, psycho‐behavioural intervention, survivorship care, symptom management

## Abstract

**Background:**

Post‐treatment colorectal cancer patients commonly experience cancer‐related fatigue (CRF), yet few interventions address this concern.

**Objectives:**

To examine the feasibility, acceptability, and preliminary effects of a psycho‐behavioural intervention on CRF and other health outcomes in this population.

**Methods:**

A single‐blinded, parallel‐group RCT was conducted with a pilot sample of 50 participants randomized to a 6‐week psycho‐behavioural intervention or usual care. Feasibility was assessed through recruitment, completion, attendance, attrition, and retention rates; acceptability via satisfaction surveys and semi‐structured interviews. Outcomes included CRF, physical activity, dietary behaviours, sleep disturbance, anxiety and depressive symptoms, assessed at baseline and within 1‐week post‐intervention.

**Results:**

Feasibility benchmarks were met: recruitment 52.6%, completion 72.0%, attendance 74.7%, attrition 6.0%, and retention 94.0%. High satisfaction and positive qualitative feedback indicated good acceptability. Repeated Measures ANOVA revealed significant group‐by‐time interaction effects favouring the intervention for reducing CRF (*F* = 6.49, *p* = 0.014; Hedges' *g* = −0.71), enhancing physical activity (*F* = 19.76, *p* < 0.001; Hedges' *g* = 1.24), improving dietary behaviours (*F* = 4.74, *p* = 0.034; Hedges' *g* = 0.61), and alleviating sleep disturbance (*F* = 9.64, *p* = 0.003; Hedges' *g* = −0.86), but not for anxiety (*F* = 3.01, *p* = 0.089; Hedges' *g* = −0.48) or depressive symptoms (*F* = 3.77, *p* = 0.058; Hedges' *g* = −0.54).

**Conclusion:**

The psycho‐behavioural intervention demonstrated feasibility, acceptability, and preliminary efficacy in reducing CRF, enhancing physical activity, improving dietary behaviours, and alleviating sleep disturbance in this population. A definitive RCT is warranted to evaluate longer‐term effects.

## 1 Background

Colorectal cancer ranks third in global cancer prevalence and second in China [1, 2]. Primary treatments involve surgery, supplemented by chemoradiotherapy [3]. Patients with stage I‐III colorectal cancer are commonly treated with curative intent and subsequently enter a defined survivorship rehabilitation phase, with reported 5‐year relative survival rates of approximately 57.0%–91.0%. Patients with stage IV disease often follow metastatic‐disease treatment trajectories involving ongoing systemic therapy, disease control, symptom palliation, and life prolongation, with a substantially lower 5‐year relative survival rate of approximately 14.0% [4]. Among the persistent symptoms reported by 32.4%–79.0% of colorectal cancer survivors, cancer‐related fatigue (CRF) is among the most common and distressing [5].

Cancer‐related fatigue is characterized by distressing persistent subjective sense of physical (e.g., bodily weakness, reduced stamina), emotional (e.g., loss of motivation, reduced self‐esteem, depressive feelings), and/or cognitive (e.g., difficulties in concentration, attention, memory, information processing) tiredness or exhaustion associated with cancer or its treatment, which is disproportional to recent activity levels and interferes with usual functioning [6, 7]. Treatment‐related sequelae predispose colorectal cancer patients to multidimensional CRF. Surgery causes bowel dysfunction (e.g., diarrhoea, incontinence, and stoma), which impairs nutrition, disrupt sleep, and restrict physical and social activities [8], collectively resulting in physical fatigue. Chemotherapy regimens commonly used in colorectal cancer, such as CAPEOX (capecitabine and oxaliplatin) and mFOLFOX6 (modified folinic acid, fluorouracil, and oxaliplatin), may induce peripheral neuropathy, gastrointestinal toxicities, and cognitive impairment that amplify physical and cognitive fatigue [9]. Additionally, stoma formation precipitates emotional fatigue through body image disturbance and social withdrawal, and concerns about leakage or odour [10]. Critically, CRF does not resolve with treatment completion; without appropriate management, it may persist or even deteriorate within 12 months after treatment [11]. Very severe CRF is associated with increased all‐cause mortality risk [12], and higher continuous CRF scores are associated with more severe anxiety and depressive symptoms [13], collectively compromising quality of life. Therefore, strategies to alleviate CRF specifically for colorectal cancer patients are warranted.

Non‐pharmacological approaches, particularly physical exercises and psychosocial interventions, represent the predominant evidence‐based strategies for managing CRF [14]. Although physical exercise has demonstrated enduring effects in reducing CRF among patients with colorectal cancer [15], barriers such as fatigue itself and physical limitations may limit sustained engagement, particularly among patients with advanced disease [16]. Psychosocial interventions, which aim to inform, educate, and enhance individuals' coping capacities in relation to illness and its consequences, may offer particular value by addressing cognitive, emotional, and behavioural processes associated with fatigue that exercise alone may not fully target [17].

Despite growing evidence on psychosocial interventions, research gaps remain in colorectal cancer. A systematic review of 41 randomized controlled trials (RCTs) on psychosocial interventions for CRF found that fewer than 10% of participants were colorectal cancer patients, limiting applicability [18]. Colorectal cancer‐specific RCTs have demonstrated the efficacy of Solution Focused Therapy (SFT) to explore patients' potential resources to build solutions; Mindfulness‐Based Stress Reduction (MBSR) to cultivate present‐moment awareness without judgement to reduce stress; and Attention and Interpretation Therapy (AIT) to help pay attention to here‐and‐now, and interpret experiences with gratitude, compassion, acceptance, forgiveness, high meaning and purpose [19]. However, these trials predominantly targeted CRF during active treatments when fatigue is driven by acute treatment‐related physiological processes and fluctuates with treatment cycles. Less attention has been given to post‐treatment CRF, which may persist beyond acute cytotoxic exposure and become maintained by interacting physical, psychological, and behavioural factors, including deconditioning, sleep disturbance, fatigue catastrophising, avoidance behaviour, and physical inactivity [20]. This gap is critical as post‐treatment survivors often lack professional support [10]. Moreover, these trials mainly employed single‐component interventions (psychological or behavioural strategies alone), overlooking the multidimensional nature of CRF, and often lacked an explicit theoretical framework specifically targeting fatigue.

To address these gaps, we conducted a systematic review and meta‐analysis of nine RCTs examining psychosocial interventions for CRF in colorectal cancer patients [19]. Six trials enroled patients during active chemotherapy and only one targeted post‐treatment survivors, confirming underrepresentation of the post‐treatment survivorship and justifying the focus of this study. The results indicated that psychosocial interventions significantly reduced CRF at short‐, medium‐, and long‐term follow‐up, with pooled effect sizes of −0.24 to −0.53, and identified effective intervention features: psycho‐behavioural approaches integrating emotion expression and behavioural techniques, delivered across six weekly 60‐min individual sessions in a blended face‐to‐face and videoconferencing mode.

**FIGURE 1:**
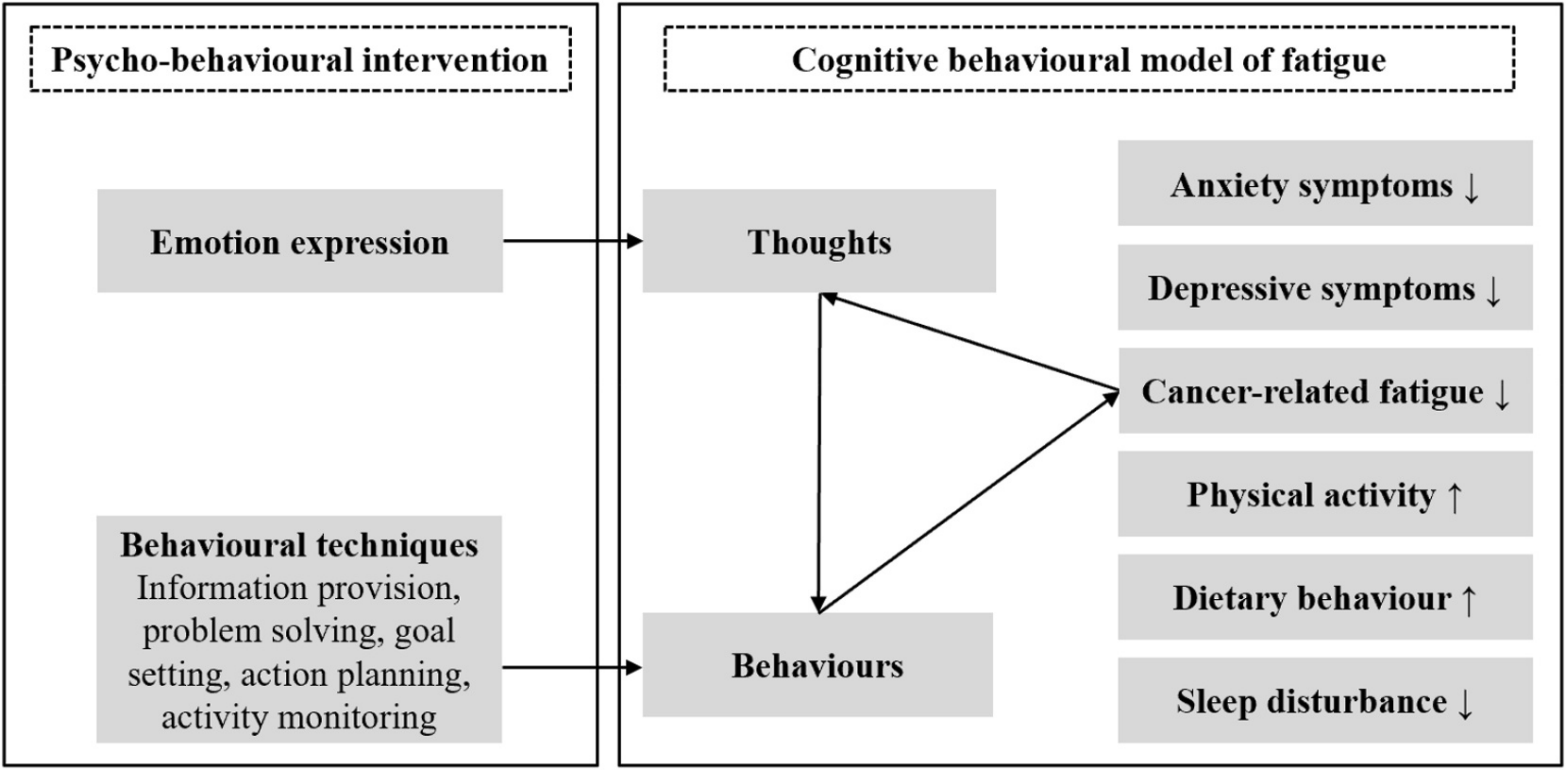
Theoretical framework of the psycho‐behavioural intervention programme.

Informed by these findings [19], cognitive behavioural model of fatigue positing that fatigue is maintained by a cycle of maladaptive thoughts and behaviours [21], and Emotion‐Focused Therapy manuals guiding the design of emotion expression [22], we developed a theory‐driven, multi‐component psycho‐behavioural intervention for post‐treatment colorectal cancer patients (Figure 1). The intervention integrates emotion expression (addressing maladaptive thoughts such as avoidance of healthy behaviours) and behavioural techniques (information provision, goal setting, action planning, activity monitoring, and problem solving) targeting physical activity, dietary behaviours, and sleep hygiene [19]. These behavioural domains are recommended for CRF management in clinical guidelines [6, 14], and commonly problematic in this population [10]. The 6‐week blended delivery mode were considered appropriate for post‐treatment patients requiring flexible, personalized care that minimizes recovery burden [19]. Accordingly, this pilot study aims to examine the feasibility, acceptability, and preliminary effects of the psycho‐behavioural intervention on CRF, physical activity, dietary behaviours, sleep disturbance, anxiety, and depressive symptoms among post‐treatment colorectal cancer patients.

## 2 Methods

### 2.1 Study Design and Settings

An assessor‐blinded, parallel pilot RCT with pre‐post measures was conducted from April to June 2025 at colorectal outpatient clinics of a tertiary cancer hospital in Jinan, Shandong Province, China. This hospital handles over 80,000 colorectal cancer outpatient visits annually, representing over 60% of regional cases. This study was guided by the Medical Research Council (MRC) framework for developing and evaluating complex interventions [23], registered with Chinese Clinical Trial Registry (ChiCTR2500104303), and reported per CONSORT extension guidelines for pilot and feasibility trials [24].

### 2.2 Participants and Sample

Based on the stepped rules of thumb [25], a pilot sample of 20 participants per arm was determined to be sufficient for estimating the parameters of a main trial powered at 80% with a small‐to‐medium target effect size. Accounting for a conservative 20% anticipated attrition rate, the initial recruitment target was 50 participants (25 per arm).

Individuals were included if they: (1) were aged ≥ 18 years; (2) had a clinical diagnosis of colorectal cancer at stage I–III (3) had completed primary treatments (e.g., surgery, chemotherapy, radiotherapy); within the past 12 months; (4) had clinically meaningful CRF, with a score of ≥ 18 on the Cancer Fatigue Scale (CFS) [26]; (5) were able to communicate in Mandarin; (6) had access to WeChat and were proficient in conducting real‐time videoconferencing via it. Stage IV patients were excluded given their distinct care priorities centred on disease control, palliation, and prolonging survival rather than survivorship rehabilitation, and their inclusion would have introduced clinical heterogeneity, limiting internal validity. Individuals were further excluded if they had a clinical diagnosis of other cancers, cognitive deficits (Abbreviated Mental Test < 8), mental disorders, or were participating in other studies.

Convenience sampling was utilised. A trained case nurse screened appointment lists and medical records to identify potential participants, who were then referred to a trained research assistant for eligibility assessment and informed consent.

### 2.3 Randomization, Allocation Concealment, and Blinding

Participants were allocated using stratified block randomization (stratified by cancer stage and sex; block sizes of four or six; 1:1 ratio) via computer‐generated lists, with allocation concealment maintained through sequentially numbered sealed opaque envelopes. Blinding of the intervener and participants was not feasible given the intervention's nature, however, outcome assessments were conducted by a trained assessor blinded to group allocation. To minimise inadvertent unblinding, participants were instructed not to disclose their group allocation or intervention‐related activities during assessments, and the assessor was trained to redirect any conversations that risked revealing allocation status.

### 2.4 Treatment Groups

Participants in the control group received usual care: a 30‐min individual face‐to‐face health education session delivered by a colorectal professional, supplemented by an information booklet outlining follow‐up protocols per Chinese Society of Clinical Oncology guidelines.

In addition to usual care, participants in the intervention group received a psycho‐behavioural intervention, consisting of six weekly, 60‐min individual sessions delivered by the principal investigator (LJL). Session 1 was conducted face‐to‐face to facilitate direct observation, contextualised therapeutic work, and rapid rapport, and Session 2–6 were delivered via videoconferencing to enhance accessibility and flexibility. Sessions integrated emotion expression, which helped clients identify illness‐related emotions and associated maladaptive thoughts, articulate emotions verbally, and reframe their emotional narrative, with behavioural techniques (information provision, problem solving, activity monitoring, goal setting, and action planning) targeting physical activity, dietary behaviours, and sleep hygiene. For example, emotion expression provided a structured outlet for stoma‐related body image disturbance. Behavioural techniques facilitated participants, especially those with stomas, to avoid gas‐producing high‐fibre foods and adopt small, frequent meal patterns; to refrain from high‐intensity or abdominal‐loading exercises to prevent stoma prolapse; and to limit food and fluid intake within 2 hours before bedtime to minimise nocturnal bowel movement demands. The theoretical and empirical basis for each component was detailed in Supporting Information S1: Table S1. Sequentially, Session 1 provided educational information and problem identification; Session 2 established goals and introduced activity monitoring; and Session 3–6 addressed emotional barriers while adapting plans for sustained behavioural engagement (Table 1). An information booklet was distributed before Session 1, covering guidance on managing treatment side effects, information on CRF, healthy physical activity, dietary behaviours, and sleep hygiene, and structured practice logs. The intervention content was validated by an expert panel of six experts (Scale‐level Content Validity Index, S‐CVI = 1.00) (Supporting Information S1: Table S2) [27], and four lay individuals from the target population confirming its comprehensibility and relevance to their survivorship needs.

**TABLE 1:** Session‐by‐session outline of the psycho‐behavioural intervention for cancer‐related fatigue management.

Theme	Intervention components	Intervention content	
		Objectives	Key activities
Session 1 Get to know cancer‐related fatigue (CRF)	Problem solving, information provision	Establish rapport Provide an intervention overview Facilitate participants to identify problems and solutions to their physical activity, dietary behaviours, and sleep hygiene Increase knowledge of colorectal cancer, CRF, physical activity, dietary behaviours, and sleep hygiene	Session orientation: Rapport building and agenda introduction Intervention overview: Highlight the features of the psycho‐behavioural intervention (e.g., rundown, dosage, modalities), and introduce the ground rules Problem solving: Encourage participants to identify problems and solutions in engaging in physical activity, dietary behaviours, and sleep hygiene using six steps of problem solving (problem identification, problem analysis, generating potential solutions, evaluating solutions, implementing the solution, and evaluating the outcome) Information provision: Guide participants through the information booklet covering colorectal cancer, CRF, physical activity, dietary behaviours, and sleep hygiene Session round‐up: Participant summary with facilitator supplements
Session 2 Introducing goal setting, action planning, and activity monitoring	Goal setting, action planning, activity monitoring	Help participants set realistic goals and formulate concrete action plans based on the problems and solutions identified in the session 1 Introduce activity monitoring to track activity progress	Session orientation: Recapping session 1 and agenda introduction Goal setting: Based on the problems and solutions identified in session 1 and the principles from the information booklet, help participants set two realistic goals about physical activity, dietary behaviours, and sleep hygiene for the following week Action planning: Collaboratively develop specific plans (what/when/where/who/how) for achieving the above goals Activity monitoring: Introduce the daily activity log to record completed activities, completion degree, and any barriers encountered Homework assignment: Encourage participants to complete scheduled activities, and to complete the daily activity log Session round‐up: Participant summary with facilitator supplements
Session 3–6 Ongoing problem solving and emotion expression	Problem solving, emotion expression, goal setting, action planning	Facilitate participants to address barriers which hinder their engagement in healthy physical activity, dietary behaviours, and sleep hygiene Facilitate participants' dysfunctional cognitive‐emotional awareness, expression, and transformation Help participants refine their action goals and plans	Session orientation: Recapping the previous session and agenda introduction Homework review: Review the daily activity log; discuss completion degree and barriers encountered; invite participants to share emotional expression experiences (sessions 4–6) Problem solving: Facilitate participants to identify the most salient barrier and relevant solutions using six steps of problem solving Emotion expression – *Emotional awareness*: Use focusing—facilitate participants to close their eyes, breathe slowly, and scan their body to locate where a difficult experience is felt physically, then prompt them to sit with that bodily sensation until a word, image, or phrase emerges to capture it—to identify and articulate current emotional experiences – *Emotion evocation and maladaptive thoughts identification*: Use empty chair exercise—invite participants to speak directly to a significant person, situation, or aspect of their experience (e.g., fatigue) imagined as sitting in an empty chair—to evoke and deepen primary emotions; and identify attached maladaptive thoughts – *Cognitive‐emotional reframing*: Use the two‐chair exercise—facilitate dialog between two conflicting aspects of self (e.g., critical vs. compassionate) by having the participant alternate between two chairs—to help participants access unmet needs, transform maladaptive emotions into adaptive ones, and construct new personal narratives Goal setting & action planning: Modify previous goals based on progress and newly resolved barriers; set two more realistic goals about physical activity, dietary behaviours, and sleep hygiene for the following week Homework assignment: Encourage participants to complete scheduled activities; to complete the daily activity log; to perform emotion expression practice when emotional barriers arise Session round‐up: Participant summary with facilitator supplements

### 2.5 Treatment Fidelity

The intervention was delivered based on the validated protocol by a PhD candidate in nursing (LJL) with 2 years of clinical experience in gastrointestinal oncology. The intervener completed Emotional‐Focused Therapy training, obtained certification, and conducted session‐by‐session case‐scenario rehearsals before programme launch. All sessions were audio‐recorded, 20% of which (23 of 112 total sessions) were randomly selected and reviewed by a researcher with expertise in colorectal cancer rehabilitation using a self‐developed checklist (Supporting Information S1: Table S3), with protocol adherence was 100%, 98.2%, and 95.7% for Session 1, 2, and 3–6, respectively. Participants received WeChat reminders one day prior to each session to enhance adherence.

### 2.6 Data Collections and Outcome Measurements

Data were collected by a research assistant blinded to group allocation and trained in standardised questionnaire administration, verbatim reading of items and response options, neutral interviewing, accurate response recording, confidentiality, and maintenance of blinding. Socio‐demographic and clinical characteristics were assessed at baseline (T0), and primary and secondary outcomes were assessed at baseline and immediately post‐intervention (T1, within 1‐week post‐intervention). At T0, data were collected face‐to‐face, with participants encouraged to self‐complete questionnaires whenever feasible. Where self‐completing was not feasible, the research assistant read each item and response options aloud and recorded verbal responses without prompting. At T1, assessments were conducted by telephone using the same standardised oral‐administration procedure.

Feasibility was evaluated using rates of recruitment, completion, attendance, attrition, and retention. At each study stage (eligibility assessment, enrolment, follow‐up), a trained research assistant systematically recorded reasons for exclusion, refusal, incomplete participation, and dropout on a structured form. Intervention completion was defined as completing at least 75% of the six sessions, equating to a minimum of five sessions.

Acceptability was evaluated among intervention participants at T1, using a self‐developed satisfaction form and individual semi‐structured interviews. The satisfaction form comprised 16 items adapted from the Chinese Standardized Client Satisfaction Scale [28], using a 5‐point Likert scale (1 = very unsatisfied, 5 = very satisfied) to assess satisfaction with intervener performance, intervention characteristics, accessibility, effects, and overall satisfaction (Supporting Information S1: Table S4). Individual semi‐structured interviews were conducted via telephone by the intervener (LJL), who had experience in qualitative interviewing, to explore participants' experience, perceived benefits, and barriers to the intervention (Supporting Information S1: Table S5).

The primary outcome was CRF, measured by Cancer Fatigue Scale, a 5‐point Likert scale (1 = no, 5 = very much; total score range: 0–60; ≥ 18 indicating clinically meaningful CRF), comprising three subscales: physical, cognitive, and emotional fatigue, with higher scores indicating greater severity [26]. Secondary outcomes included anxiety symptoms (Generalized Anxiety Disorder Questionnaire‐7), depressive symptoms (Patient Health Questionnaire‐9), physical activity and dietary behaviours (physical activity and nutrition subscales of the Health‐Promoting Lifestyle Profile‐II), and sleep disturbance (Patient‐Reported Outcomes Measurement Information System Sleep Disturbance‐Short Form 4a). All measurements were validated in Chinese cancer patients and demonstrated good reliability (Cronbach's α, 0.71–0.91). Detailed psychometric description is shown in Supporting Information S1: Table S6.

### 2.7 Data Analysis

Data were analysed using IBM SPSS 29.0 (significance level: 0.05, two‐tailed) on an intention‐to‐treat (ITT) basis, with last observation carried forward (LOCF) to address missing data. Continuous data were checked for normal distribution using Q‐Q plots and skewness and kurtosis statistics. Baseline comparisons used independent *t* tests for continuous variables and Chi‐square/Fisher's exact tests for categorical variables. Group × time interaction effects on outcomes were examined by Repeated Measures ANOVA. Effect sizes were estimated using Hedges' *g* at T1, and were interpreted as small (0.2 ≤ *g* < 0.5), medium (0.5 ≤ *g* < 0.8), and large (*g* ≥ 0.8) [29]. Interviews were audio‐recorded, transcribed verbatim, and analysed using conventional content analysis. Two researchers independently coded the data and resolved disagreements via consultation with the corresponding author. Details on qualitative data analysis are shown in Supporting Information S1: Table S5.

### 2.8 Ethical Considerations

The study adhered the principles of the Declaration of Helsinki and received approval from both The Joint Chinese University of Hong Kong — New Territories East Cluster Clinical Research Ethics Committee (No: 2025.183‐T) and the ethics committee of the research hospital (No: SDTHEC202503193). Participants provided written informed consent prior to randomisation and were informed of their rights on anonymity, confidentiality, and the potential benefits and risks of participation.

## 3 Results

### 3.1 Characteristics of Participants

As shown in Table 2, the mean age was 58.74 years (SD: 9.39), with 66% of participants being male. Colon and rectal cancer each accounted for nearly half, with 52% at stage III and the remaining at stage I–II. On average, the participants had completed treatments 6.72 months prior (SD: 3.65). The baseline mean CFS score was 35.92 (SD: 4.14). No significant between‐group differences were observed at baseline (*p* = 0.051–1.000).

**TABLE 2:** Comparison of baseline sociodemographic and clinical characteristics and outcome variables between the two groups (*n* = 50).

Characteristics	Total (*n* = 50) Mean ± SD/*n* (%)	Intervention group (*n* = 25) Mean ± SD/*n* (%)	Control group (*n* = 25) Mean ± SD/*n* (%)	*p*
Sociodemographic characteristics				
Age (years)	58.74 ± 9.39	57.52 ± 10.46	59.96 ± 8.23	0.364
Sex				0.370
Male	33 (66.0)	18 (72.0)	15 (60.0)	
Female	17 (34.0)	7 (28.0)	10 (40.0)	
Marital status				1.000
Married	48 (96.0)	24 (96.0)	24 (96.0)	
Single/divorced/widowed	2 (4.0)	1 (4.0)	1 (4.0)	
Educational level				0.138
Primary school or below	10 (20.0)	2 (8.0)	8 (32.0)	
Junior high school	19 (38.0)	11 (44.0)	8 (32.0)	
Senior high school	9 (18.0)	4 (16.0)	5 (20.0)	
College or above	12 (24.0)	8 (32.0)	4 (16.0)	
Employment status				0.051
Unemployment	4 (8.0)	0 (0.0)	4 (16.0)	
Retired	30 (60.0)	14 (56.0)	16 (64.0)	
On‐the‐job	16 (32.0)	11 (44.0)	5 (20.0)	
Monthly income (CNY)				0.441
> 5000	7 (14.0)	4 (16.0)	3 (12.0)	
3000–5000	16 (32.0)	10 (40.0)	6 (24.0)	
1000–3000	17 (34.0)	8 (32.0)	9 (36.0)	
< 1000	10 (20.0)	3 (12.0)	7 (28.0)	
Medical insurance				0.569
Urban and rural resident basic medical insurance	28 (56.0)	13 (52.0)	15 (60.0)	
Urban employee basic medical insurance	22 (44.0)	12 (48.0)	10 (40.0)	
Place of residence				0.156
Unban area	27 (54.0)	16 (64.0)	11 (44.0)	
Rural area	23 (46.0)	9 (36.0)	14 (56.0)	
Living arrangements				1.000
Living with others	49 (98.0)	24 (96.0)	25 (100.0)	
Living alone	1 (2.0)	1 (4.0)	0 (0.0)	
Clinical characteristics				
Body mass index (kg/m^2^)^a^				0.530
Underweight	0 (0.0)	0 (0.0)	0 (0.0)	
Normal weight	18 (36.0)	8 (32.0)	10 (40.0)	
Overweight	28 (56.0)	14 (56.0)	14 (56.0)	
Obesity	4 (8.0)	3 (12.0)	1 (4.0)	
Cancer diagnosis				
Colon cancer	23 (46.0)	12 (48.0)	11 (44.0)	0.777
Rectal cancer	27 (54.0)	13 (52.0)	14 (56.0)	
Cancer stage				0.503
I	8 (16.0)	3 (12.0)	5 (20.0)	
II	16 (32.0)	7 (28.0)	9 (36.0)	
III	26 (52.0)	15 (60.0)	11 (44.0)	
Time since treatment completion (months)	6.72 ± 3.65	6.88 ± 3.89	6.56 ± 3.47	0.760
Treatments have received				0.366
Surgery	7 (14.0)	3 (12.0)	4 (16.0)	
Surgery + neoadjuvant chemoradiotherapy	8 (16.0)	2 (8.0)	6 (24.0)	
Surgery + adjuvant chemoradiotherapy	27 (54.0)	16 (64.0)	11 (44.0)	
Surgery + neoadjuvant chemoradiotherapy + adjuvant chemoradiotherapy	8 (16.0)	4 (16.0)	4 (16.0)	
Stoma status				0.932
None	33 (66.0)	16 (64.0)	17 (68.0)	
Temporary stoma	9 (18.0)	5 (20.0)	4 (16.0)	
Permanent stoma	8 (16.0)	4 (16.0)	4 (16.0)	
Number of comorbidities				0.842
0	28 (56.0)	15 (60.0)	13 (52.0)	
1–3	20 (40.0)	9 (36.0)	11 (44.0)	
≥ 4	2 (4.0)	1 (4.0)	1 (4.0)	
Haemoglobin (g/L)^b^				0.864
Low	11 (22.0)	5 (20.0)	6 (24.0)	
Normal	34 (68.0)	17 (68.0)	17 (68.0)	
High	5 (10.0)	3 (12.0)	2 (8.0)	
Albumin (g/L)^c^				1.000
Normal	6 (12.0)	3 (12.0)	3 (12.0)	
Low	44 (88.0)	22 (88.0)	22 (88.0)	
High	0 (0.0)	0 (0.0)	0 (0.0)	
Outcome variables				
CRF	35.92 ± 4.14	35.28 ± 4.73	36.56 ± 3.44	0.279
Physical fatigue	15.76 ± 2.80	15.40 ± 3.08	16.12 ± 2.49	0.368
Cognitive fatigue	7.26 ± 2.46	7.00 ± 2.57	7.52 ± 2.38	0.461
Emotional fatigue	12.90 ± 1.94	12.88 ± 2.03	12.92 ± 1.89	0.943
Physical activity	14.62 ± 2.19	14.92 ± 2.69	14.32 ± 1.55	0.340
Dietary behaviours	18.58 ± 2.56	19.12 ± 3.27	18.04 ± 1.43	0.140
Sleep disturbance	48.47 ± 4.62	48.90 ± 5.41	48.03 ± 3.72	0.508
Anxiety symptoms	6.60 ± 3.64	6.48 ± 3.25	6.72 ± 4.05	0.818
Depressive symptoms	6.74 ± 3.04	7.08 ± 3.03	6.40 ± 3.07	0.434

Abbreviation: SD: standard deviation.

^a^ BMI was categorised according to the World Health Organisation (WHO) classification: underweight (< 18.5 kg/m^2^), normal weight (18.5–24.9 kg/m^2^), overweight (25.0–29.9 kg/m^2^), and obesity (≥ 30 kg/m^2^).

^b^ Haemoglobin level was categorised based on the WHO criteria: low haemoglobin level (< 120 g/L for women, and < 135 g/L for men), normal haemoglobin level (120–155 g/L for women, and 135–175 g/L for men), and high haemoglobin level (> 155 g/L for women, and > 175 g/L for men).

^c^ Albumin level was categorised as low albumin level (< 35 g/L), normal albumin level (35–54 g/L), and high albumin level (> 54 g/L).

### 3.2 Feasibility

**FIGURE 2:**
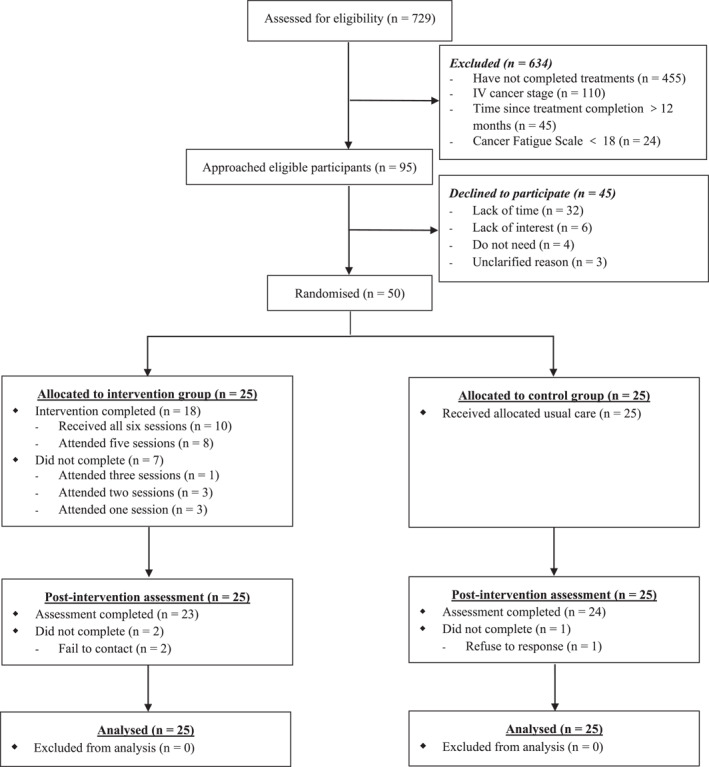
The flow chart of the pilot study.

Figure 2 shows the flow chart. Of 729 participants screened, 95 were eligible and 50 were randomized (25 per arm), yielding a recruitment rate of 52.6%. In the intervention group, 18 of 25 participants (72.0%) completed the intervention (10 attended all six sessions; 8 attended five), with an attendance rate of 74.7%. Of all 50 participants, 47 completed the post‐intervention assessment (attrition 6.0%; retention 94.0%). No adverse events were reported.

### 3.3 Acceptability

Among the 23 participants who completed the satisfactory form, 65.22% reported being very satisfied and 34.78% satisfied overall. Satisfaction was high across intervener performance (78.26%–86.96% very satisfied across items), intervention characteristics (60.87%–78.26%), accessibility (60.87%–82.61%), and intervention effects (78.26%–91.30%).

Eleven of 25 participants completed the qualitative interviews. Reasons for non‐participation included being uncontactable (*n* = 3), refusal to participate (*n* = 4), and time constraints (*n* = 7). Three main categories emerged: (1) Addressing unmet support and information needs after treatment: participants valued weekly professional support and new understanding of CRF and symptom management. (2) Developing practical skills for physical and emotional challenges: participants reported the intervention helped translate complex wellness goals into achievable behavioural steps and provided emotion regulation techniques applicable to real‐life stressors. (3) Suggestions for improving intervention delivery: participants suggested providing a digital version of the information booklet, offering an online option for the first session to reduce scheduling conflicts, and sending session reminders earlier. Detailed description on these categories, supported with quotes, is shown in Supporting Information S1: Table S7.

### 3.4 Preliminary Effects

Baseline comparisons revealed no significant between‐group differences across all outcome measures at T0 (*p* = 0.140–0.943) (Table 2). The Repeated Measures ANOVA demonstrated significant group‐by‐time interaction effects favouring the intervention group for reducing total CRF (*F* = 6.49, *p* = 0.014), physical fatigue (*F* = 14.01, *p* < 0.001), and emotional fatigue (*F* = 4.87, *p* = 0.032), with moderate to large effect sizes (Hedges' *g* ranging from −0.61 to −1.05) (Table 3). Additionally, the intervention demonstrated significant interaction effects on enhancing physical activity (*F* = 19.76, *p* < 0.001; Hedges' *g* = 1.24), improving dietary behaviours (*F* = 4.74, *p* = 0.034; Hedges' *g* = 0.61), and alleviating sleep disturbance (*F* = 9.64, *p* = 0.003; Hedges' *g* = −0.86). However, no significant interaction effects were observed for cognitive fatigue, anxiety and depressive symptoms (*p* = 0.058–0.376).

**TABLE 3:** Comparison of baseline (T0) and immediate post‐intervention (T1) outcome variables between two groups using Repeated Measures ANOVA (*n* = 50).

Outcome variables	Timepoint	Intervention group (*n* = 25) Mean ± SD	Control group (*n* = 25) Mean ± SD	Group effect		Time effect		Interaction effect		Hedges' *g* (95% CI)
				*F*	*p*	*F*	*p*	*F*	*p*	
Cancer‐related fatigue	T0	35.28 ± 4.73	36.56 ± 3.44	4.45	0.04	13.06	< 0.001	6.49	0.014	−0.71 (−1.28, −0.14)
	T1	33.20 ± 3.72	36.20 ± 3.03							
Physical fatigue	T0	15.40 ± 3.08	16.12 ± 2.49	3.11	0.084	32.93	< 0.001	14.01	< 0.001	−1.05 (−1.64, −0.45)
	T1	13.88 ± 2.96	15.8 ± 2.20							
Emotional fatigue	T0	12.88 ± 2.03	12.92 ± 1.89	0.68	0.414	14.07	< 0.001	4.87	0.032	−0.61 (−1.18, −0.05)
	T1	11.80 ± 2.10	12.64 ± 1.96							
Cognitive fatigue	T0	7.00 ± 2.57	7.52 ± 2.38	0.36	0.551	5.89	0.019	0.80	0.376	0.25 (−0.31, 0.81)
	T1	7.52 ± 2.35	7.76 ± 1.85							
Physical activity	T0	14.92 ± 2.69	14.32 ± 1.55	5.88	0.019	23.61	< 0.001	19.76	< 0.001	1.24 (0.63, 1.85)
	T1	16.72 ± 2.76	14.40 ± 1.66							
Dietary behaviours	T0	19.12 ± 3.27	18.04 ± 1.43	7.44	0.009	3.23	0.078	4.74	0.034	0.61 (0.04, 1.17)
	T1	19.96 ± 2.47	17.96 ± 1.62							
Sleep disturbance	T0	48.90 ± 5.41	48.03 ± 3.72	0.01	0.917	293.09	< 0.001	9.64	0.003	−0.86 (−1.44, −0.28)
	T1	44.04 ± 4.75	44.66 ± 3.89							
Anxiety symptoms	T0	6.48 ± 3.25	6.72 ± 4.05	0.71	0.403	46.84	< 0.001	3.01	0.089	−0.48 (−1.05, 0.08)
	T1	4.60 ± 2.16	5.60 ± 3.15							
Depressive symptoms	T0	7.08 ± 3.03	6.40 ± 3.07	0.17	0.678	11.36	0.001	3.77	0.058	−0.54 (−1.11, 0.03)
	T1	6.04 ± 1.97	6.12 ± 2.30							

Abbreviations: CI: confidence interval; *F* = Repeated Measures ANOVA statistic; SD: standard deviation.

## 4 Discussion

This pilot RCT evaluated the feasibility, acceptability and preliminary efficacy of a psycho‐behavioural intervention for post‐treatment colorectal cancer patients. Overall, the intervention was feasible and acceptable. Preliminary efficacy analyses suggested significant improvements in CRF, physical activity, dietary behaviours, and sleep disturbance, with moderate‐to‐large effect sizes, whereas no significant changes were observed in cognitive fatigue, anxiety, and depressive symptoms.

### 4.1 Feasibility and Acceptability

The intervention met established feasibility benchmarks for pilot trials. The attrition rate (6.0%) was comparable to previous psychosocial interventions in patients with colorectal cancer (5.5%–15.4%) [19]. The recruitment rate (52.6%) was slightly lower than prior trials (79.4%–95.7%) [19], largely because 32 of 95 eligible participants declined due to conflicts between the mandatory face‐to‐face first session and scheduled medical examinations. Both the attendance (74.7%) and completion rates (72.0%) met the commonly recommended ≥ 70% threshold for pilot feasibility trials [30], and the retention rate (94.0%) substantially exceeded the anticipated 80% threshold.

By integrating qualitative process evaluation with quantitative feasibility indicators, this study provides richer insights than previous trials, which rarely incorporated qualitative methods [19]. Qualitative findings revealed that the intervention addressed participants' unmet needs for post‐treatment professional support and information while equipping them with practical behavioural and emotional regulation skills. These features contributed to high perceived acceptability, corroborated by quantitative data showing 91.30% of participants reporting very high satisfaction with improvements in healthy behaviours and emotional coping. Additionally, the three refinement suggestions targeted intervention delivery features rather than core content, further confirming acceptability of the intervention itself. Incorporating these optimizations in future trials may improve recruitment efficiency and enhance accessibility in this population.

### 4.2 Preliminary Efficacy

Although the pilot study was not powered to provide definitive conclusions, the results provided preliminary evidence supporting the efficacy of the psycho‐behavioural intervention. Significant group‐by‐time interaction effects favouring the intervention group were observed for reducing total CRF, physical fatigue, and emotional fatigue, with moderate to large effect sizes (Hedges' *g* = −0.61 to −1.05). These findings are consistent with our previous systematic review (SMD = −0.60) [19], and another review in mixed‐cancer populations (SMD = −0.84 to −1.23) [18]. The reduction in total CRF could be theoretically explained by the cognitive behavioural model of fatigue [21]: the intervention may have disrupted self‐reinforcing fatigue cycles by simultaneously addressing maladaptive thoughts via emotion expression and behavioural contributors via behavioural techniques.

Reductions in physical fatigue may be primarily ascribed to behavioural components targeting physical activity, dietary behaviours, and sleep hygiene, which translated the overwhelming behavioural goals into achievable steps. Increased physical activity improves cardiorespiratory fitness, skeletal muscle metabolism, and neuroendocrine regulation [31]; healthier diet patterns support energy homeostasis and reduce systemic inflammation [32]; and improved sleep hygiene may restore circadian regulation and reduce fatigue perpetuation [33]. Collectively, these improvements enhance overall energy balance and thereby alleviate physical fatigue. The significant reduction in emotional fatigue may be explained through two complementary ways. Directly, emotion expression facilitated emotional processing by encouraging participants to articulate and cognitively reappraise cancer‐related stressors, thereby reducing emotional suppression and psychological burden [34]. Indirectly, improvements in those behavioural domains may have enhanced emotional well‐being: regular physical activity is associated with improved mood through endorphin release and serotonergic regulation [35], while improved sleep quality may restore prefrontal regulation of emotional processes [36]. Both pathways may act synergistically to reduce emotional fatigue. The intervention did not produce significant effects on cognitive fatigue, possibly due to floor effects from relatively low baseline levels, the need for longer follow‐up, and limited statistical power in this pilot sample.

Significant effects were also observed for physical activity (Hedges' *g* = 1.24), dietary behaviours (Hedges' *g* = 0.61), and sleep disturbance (Hedges' *g* = −0.86), suggesting that behavioural strategies and emotion expression techniques effectively enhanced participants' engagement in these healthy behaviours. These domains may represent proximal mechanisms through which the intervention reduces CRF, and future research should formally examine these mediational pathways.

Conversely, no significant effects were observed for anxiety and depressive symptoms, contrasting with previous systematic reviews which reported beneficial effects of psychosocial interventions on depressive symptoms among colorectal and breast cancer patients [19, 37]. Given that CRF and depressive symptoms share overlapping manifestations [38], and that CRF, anxiety, and depressive symptoms tend to co‐occur as a symptom cluster with bidirectional interactions in colorectal cancer [39], the non‐significant findings may be attributable to floor effects from low baseline scores, the need for longer follow‐up as effects on depressive symptoms typically emerge at 3 months or later [19], and insufficient statistical power. Future adequately powered RCTs with longer follow‐up are warranted.

### 4.3 Clinical Implications

These findings provide several implications for survivorship care. First, healthcare professionals should routinely screen post‐treatment colorectal cancer survivors for CRF, with confirmed cases receiving timely and targeted management. Second, the significant improvements observed across CRF, physical activity, dietary behaviours, and sleep disturbance indicate that the multi‐component psycho‐behavioural approaches may be more appropriate than single‐component approaches given the multi‐dimensional nature of CRF. Third, incorporating videoconferencing delivery into the programme provides a practical and accessible modality to reduce patient burden while maintaining therapeutic quality. Fourth, qualitative findings demonstrate survivors' unmet needs to professional support, highlighting the importance of integrating structured psycho‐behavioural interventions into routine care. In this regard, training supervised paraprofessionals (e.g., nurses) to deliver such interventions may enhance scalability and reduce strain on specialist resources.

### 4.4 Strengths and Limitations

This study presents several strengths. First, the rigorous methodological design, including stratified block randomization based on cancer stage and sex, ensured balanced group distribution and minimized selection bias. Second, the intervention was systematically developed following the MRC framework for complex interventions grounded in the cognitive behavioural model of fatigue. Third, the focus on post‐treatment patients filled a research gap, providing valuable insights into survivorship care for this often‐underserved population. Finally, the study employed mixed‐methods assessment, providing a comprehensive evaluation of feasibility, acceptability, and preliminary effects.

Several limitations warrant consideration. First, the small single‐centre sample limited the internal and external validity of the study. Second, the absence of follow‐up assessments limited the interpretation of sustained intervention effects. Future definitive trials should incorporate longer follow‐up assessments (e.g., 3 months) to evaluate sustainability of treatment effects. Third, reliance on a self‐developed intervention fidelity checklist may introduce subjective and reporting biases. Fourth, although the intervention attempted to minimize staffing and resource burden, further optimization is possible, such as training supervised paraprofessionals to facilitate sessions. Fifth, due to insufficient statistical power inherent to this pilot study, outcomes (CRF, anxiety, and depressive symptoms) were analysed only as continuous variables. Future definitive RCTs should incorporate categorical analyses based on clinical cutoffs to capture clinically meaningful changes. Sixth, although the outcome assessor was blinded to group allocation with procedural safeguards in place, blinding integrity was not formally verified, leaving the possibility of inadvertent unblinding undetected. Finally, missing outcome data were handled using LOCF, which, while acceptable in pilot studies, assumes static outcomes after dropout and may bias effect estimates. Future definitive trials should adopt more principled approaches, such as multiple imputation under a missing‐at‐random (MAR) assumption or mixed‐effects models for repeated measures (MMRM), to make better use of available data [40].

## 5 Conclusion

This pilot study supports the feasibility and acceptability of the psycho‐behavioural intervention for post‐treatment colorectal cancer patients. Preliminary findings indicate significant effects on reducing CRF, enhancing physical activity, improving dietary behaviours, and alleviating sleep disturbance. Minor modifications on intervention delivery are recommended to enhance engagement and adherence. Full‐scale RCTs with longer follow‐up are warranted to confirm these effects and evaluate their long‐term sustainability.

## Author Contributions

**Jinling Lu:** writing – original draft, writing – review and editing, validation, resources, project administration, methodology, investigation, formal analysis, data curation, conceptualization. **Yuen Yu Chong:** writing – review and editing, supervision, methodology, conceptualization.

## Funding

The authors have nothing to report.

## Conflicts of Interest

The authors declare no conflicts of interest.

## Supporting information


Supporting Information S1

## Data Availability

The data that support the findings of this study are available from the corresponding author upon reasonable request.
