# Supplementary material for: Effects of a Psycho‐Behavioural Intervention on Cancer‐Related Fatigue Among Patients With Colorectal Cancer: A Pilot Randomised Controlled Trial
Source: Psychooncology. 2026 Aug 7;35(8):e70570. doi: 10.1002/pon.70570 (PMC13451782; doi:10.1002/pon.70570)
Supplement: Supplementary file 1 [file PON-35-e70570-s001.docx]

**Supplementary Table S1.** The theoretical and empirical basis for intervention components

| **Evidence sources** | **Intervention components** | **Informed procedures** |
| --- | --- | --- |
| Systematic review | Emotion expression, information provision, goal setting, action planning, problem solving, activity monitoring | The systematic review identified the optimal intervention approaches for reducing CRF in patients with colorectal cancer, that is psycho-intervention interventions integrating emotion expression and behavioural techniques (information provision, goal setting, action planning, problem solving, activity monitoring). |
| Cognitive behavioural model of fatigue | Emotion expression, information provision, goal setting, action planning, problem solving, activity monitoring | The cognitive behavioural model of fatigue, which posits that fatigue is maintained by a cycle of maladaptive thoughts and behaviours, provides a theoretical framework for psycho-behavioural interventions, with emotion expression targeting maladaptive thoughts and those behavioural techniques targeting maladaptive behaviours. |
| Emotion-Focused Therapy manuals | Emotion expression | The Emotion-Focused Therapy manuals informed the development of emotion expression, guiding the selection of the focusing exercise, empty chair exercise, and two-chair exercise to facilitate the participants to recognise and transform maladaptive emotions and thoughts into adaptive ones. |
| American Cancer Society Colorectal Cancer Survivorship Care Guideline | Information provision | The American Cancer Society Colorectal Cancer Survivorship Care Guideline informed the educational content on colorectal cancer and treatment side effects management strategies. |
| National Comprehensive Cancer Network Guideline for Survivorship Care for Healthy Living | Information provision, goal setting, action planning, problem solving, activity monitoring | The National Comprehensive Cancer Network Guideline for Survivorship Care for Healthy Living informed the educational content on healthy physical activity and dietary behaviours (information provision). Based on this knowledge, participants were facilitated to identify their problems and barriers in engaging in healthy physical activity and dietary behaviours (problem solving), and to set realistic goals and relevant plans (goal setting and action planning), along with tracking their behavioural progress (activity monitoring). |
| National Comprehensive Cancer Network Guideline for Cancer-Related Fatigue | Information provision, goal setting, action planning, problem solving, activity monitoring | The National Comprehensive Cancer Network Guideline for Cancer-Related Fatigue informed the educational content on CRF and healthy sleep hygiene (information provision). Similarly, based on this knowledge, participants were facilitated to identify their problems and barriers in engaging in healthy sleep hygiene (problem solving), and to set realistic goals and relevant plans (goal setting and action planning), along with tracking their behavioural progress (activity monitoring). |

**Supplementary Table S2.** Content validation questionnaire

This questionnaire is designed to evaluate the content validity of a psycho-behavioural intervention that aims at reducing cancer-related fatigue (CRF) in post-treatment patients with colorectal cancer. It encompasses all six sessions of the intervention. You are kindly requested to assess each session's content's clarity, relevance, and adequacy.

“Clarity” evaluates how comprehensible the content is, ensuring it's presented in a straightforward and understandable manner. “Relevance” measures the extent to which the content aligns with the needs of the post-treatment patients with colorectal cancer. “Adequacy” determines whether the content thoroughly and sufficiently addresses the intended objectives.

The assessment utilises a 4-point Likert scale, where 1 = "Very unclear/totally irrelevant/highly inadequate", 2 = "Unclear/irrelevant/inadequate", 3 = "Clear/relevant/adequate", and 4 = "Very clear/highly relevant/very adequate".

| **Session** | **Content** | **Clarity** | | | | **Relevance** | | | | **Adequacy** | | | | **Suggestions for improvement** |
| --- | --- | --- | --- | --- | --- | --- | --- | --- | --- | --- | --- | --- | --- | --- |
| **1** | Session orientation & intervention overview | 1 | 2 | 3 | 4 | 1 | 2 | 3 | 4 | 1 | 2 | 3 | 4 |  |
|  | Problem solving | 1 | 2 | 3 | 4 | 1 | 2 | 3 | 4 | 1 | 2 | 3 | 4 |  |
|  | Information provision | 1 | 2 | 3 | 4 | 1 | 2 | 3 | 4 | 1 | 2 | 3 | 4 |  |
| **Session** | **Content** | **Clarity** | | | | **Relevance** | | | | **Adequacy** | | | | **Suggestions for improvement** |
| **2** | Session orientation | 1 | 2 | 3 | 4 | 1 | 2 | 3 | 4 | 1 | 2 | 3 | 4 |  |
|  | Goal setting | 1 | 2 | 3 | 4 | 1 | 2 | 3 | 4 | 1 | 2 | 3 | 4 |  |
|  | Action planning | 1 | 2 | 3 | 4 | 1 | 2 | 3 | 4 | 1 | 2 | 3 | 4 |  |
|  | Activity monitoring | 1 | 2 | 3 | 4 | 1 | 2 | 3 | 4 | 1 | 2 | 3 | 4 |  |
|  | Homework assignment | 1 | 2 | 3 | 4 | 1 | 2 | 3 | 4 | 1 | 2 | 3 | 4 |  |
|  | Session round-up | 1 | 2 | 3 | 4 | 1 | 2 | 3 | 4 | 1 | 2 | 3 | 4 |  |
| **Session** | **Content** | **Clarity** | | | | **Relevance** | | | | **Adequacy** | | | | **Suggestions for improvement** |
| **3-6** | Session orientation | 1 | 2 | 3 | 4 | 1 | 2 | 3 | 4 | 1 | 2 | 3 | 4 |  |
|  | Homework review | 1 | 2 | 3 | 4 | 1 | 2 | 3 | 4 | 1 | 2 | 3 | 4 |  |
|  | Goal setting | 1 | 2 | 3 | 4 | 1 | 2 | 3 | 4 | 1 | 2 | 3 | 4 |  |
|  | Action planning | 1 | 2 | 3 | 4 | 1 | 2 | 3 | 4 | 1 | 2 | 3 | 4 |  |
|  | Problem solving | 1 | 2 | 3 | 4 | 1 | 2 | 3 | 4 | 1 | 2 | 3 | 4 |  |
|  | Emotion expression | 1 | 2 | 3 | 4 | 1 | 2 | 3 | 4 | 1 | 2 | 3 | 4 |  |
|  | Homework assignment | 1 | 2 | 3 | 4 | 1 | 2 | 3 | 4 | 1 | 2 | 3 | 4 |  |
|  | Session round-up | 1 | 2 | 3 | 4 | 1 | 2 | 3 | 4 | 1 | 2 | 3 | 4 |  |

**Information of the assessor**

Gender: Age: Highest level of education:

Specialty: Job title: Years of working in the specialty:

**Expert Authority Questionnaire**

1. Your familiarity with the survey content (self-assessment by the expert, please mark “√” in the appropriate box):

🞎Very familiar	🞎Quite familiar	🞎Moderately familiar

🞎Slightly familiar	🞎Not familiar

2. Your basis for judgment and the degree of influence (please mark “√” in the appropriate column):

| Basis for judgement | Degree of influence | | |
| --- | --- | --- | --- |
|  | High | Medium | Low |
| Theoretical analysis |  |  |  |
| Practical experience |  |  |  |
| Reference to literature |  |  |  |
| Intuition |  |  |  |

**Supplementary Table S3.** Intervention fidelity checklist

**Session 1**

Participant ID: _________ Intervention date: _________ Intervention duration: _________

Below are listed a number of statements. Please check whether each statement is conducted when the intervention provider is delivering intervention, by filling ‘Yes’ in the spaces provided if it is conducted, otherwise ‘No.’

| **Intervention components** | **Statements for checking** | **Yes / No** |
| --- | --- | --- |
| Problem solving | - The intervention provider helps participant to identify problems about physical activity, dietary behaviours, and sleep hygiene. |  |
|  | - The intervention provider helps participant to analyse the identified problems. |  |
|  | - The intervention provider helps participants generate potential solutions to problems as much as possible. |  |
|  | - The intervention provider helps participants evaluate the pros and cons of the proposed solutions and select the optimal one. |  |
|  | - The intervention provider helps participants work on detailed plan about the selected solution. |  |
|  | - The intervention provider requests the participant to assess her/his problems (e.g., physical activity, dietary behaviours, and sleep hygiene) following the completion of the plans about the solution. |  |
| Information provision | - The intervention provider encourages the participant to go through the information about colorectal cancer, cancer-related fatigue, physical activity, dietary behaviours, and sleep hygiene in the information booklet. |  |
|  | - The intervention provider asks participants whether they have questions about the above information and give professional advice when needed. |  |

**Session 2**

Participant ID: _________ Intervention date: _________ Intervention duration: _________

Below are listed a number of statements. Please check whether each statement is conducted when the intervention provider is delivering intervention, by filling ‘Yes’ in the spaces provided if it is conducted, otherwise ‘No.’

| **Intervention components** | **Statements for checking** | **Yes / No** |
| --- | --- | --- |
| Goal setting | - The intervention provider helps participant to set one to two realistic goals for the next week, which are specific and measurable, according to the problems identified in the first session. |  |
| Action planning | - The intervention provider encourages the participant to plan exactly about what/when/who/where/how to perform physical activity, dietary behaviours, and sleep hygiene for the next week, based on the above goals. |  |
| Activity monitoring | - The intervention provider explains what activity monitoring is: to record daily activities about physical activity, dietary behaviours, and sleep hygiene. |  |
|  | - The intervention provider explains why activity monitoring is needed: (1) to know what have been done in terms of physical activity, dietary behaviours, and sleep hygiene; and (2) to record any barriers encountered in engaging in activities, so as to recognise and address them accurately. |  |
|  | - The intervention provider explains how activity monitoring is performed: taking one day last week as an example, (1) to demonstrate how to perform activity monitoring in a daily activity log; and (2) to encourage daily record because it is challenging to recall activities over time. |  |

**Session 3-6**

Participant ID: _________ Intervention date: _________ Intervention duration: _________

Below are listed a number of statements. Please check whether each statement is conducted when the intervention provider is delivering intervention, by filling ‘Yes’ in the spaces provided if it is conducted, otherwise ‘No.’

| **Intervention components** | **Statements for checking** | **Yes / No** |
| --- | --- | --- |
| Goal setting | - The intervention provider helps participant to set one to two specific goals for the next week. |  |
| Action planning | - The intervention provider encourages the participant to plan exactly about what/when/who/where/how to schedule activities for the next week based on the above goals. |  |
| Problem solving | - The intervention provider helps participant to identify barriers about their engagement of physical activity, dietary behaviours, and sleep hygiene based on activity daily log. |  |
|  | - The intervention provider helps participant to analyse the identified barriers. |  |
|  | - The intervention provider helps participants generate potential solutions to barriers as much as possible. |  |
|  | - The intervention provider helps participants evaluate the pros and cons of the proposed solutions and select the optimal one. |  |
|  | - The intervention provider helps participants work on detailed plan about the selected solution. |  |
|  | - The intervention provider requests the participant to assess her/his barriers following the completion of the solution. |  |
| Emotion expression | - The intervention provider helps the participant be aware of emotions. |  |
|  | - The intervention provider facilitates the participant to evoke and deepen primary emotions (empty chair exercise). |  |
|  | - The intervention provider facilitates the participant to identify maladaptive thoughts attached to emotions. |  |
|  | - The intervention provider facilitates the participant to access unmet needs and transform new adaptive emotions (two-chair exercise). |  |
|  | - The intervention provider facilitates the participant to create new narratives. |  |

**Supplementary Table S4.** Intervention satisfaction form

The satisfaction form uses a 5-point Likert scale (1 = very unsatisfied, 5 = very satisfied) to assess satisfaction across five domains: intervener performance (items 1-4, evaluating the intervener's attitude, competence, ability to deliver easily understandable content, and ability to listen and understand participants' concerns), intervention characteristics (items 5-8, evaluating intervention content, delivery mode, format, and dose), intervention accessibility (items 9-12, evaluating ease of access, procedural efficiency, scheduling flexibility, and content readability), intervention effects (items 13-15, evaluating effects on knowledge enhancement, behavioural engagement, and cognitive coping), and overall satisfaction (item 16), as shown below:

Instruction: Below are statements regarding your experience with the psycho-behavioural intervention. Please indicate your level of satisfaction by marking “√” on the provided 5-point Likert scale. Respond in a manner that accurately reflects your experience.

| **Items** | **Very unsatisfied** | **Unsatisfied** | **Neutral** | **Satisfied** | **Very satisfied** |
| --- | --- | --- | --- | --- | --- |
| **Performance of Intervener** | | | | | |
| 1. The intervener shows kindness towards me. | 1 | 2 | 3 | 4 | 5 |
| 2. The intervener is competent and knowledgeable. | 1 | 2 | 3 | 4 | 5 |
| 3. The intervener is able to deliver services that are easy to understand. | 1 | 2 | 3 | 4 | 5 |
| 4. The intervener is able to listen to and understand my concerns. | 1 | 2 | 3 | 4 | 5 |
| **Intervention Characteristics** | | | | | |
| 5. The content of the intervention is appropriate. | 1 | 2 | 3 | 4 | 5 |
| 6. The delivery mode of the intervention is appropriate. | 1 | 2 | 3 | 4 | 5 |
| 7. The format of the intervention is appropriate. | 1 | 2 | 3 | 4 | 5 |
| 8. The duration and frequency of the intervention are appropriate. | 1 | 2 | 3 | 4 | 5 |
| **Intervention Accessibility** | | | | | |
| 9. The intervention is easily accessible. | 1 | 2 | 3 | 4 | 5 |
| 10. The intervention procedure is efficient. | 1 | 2 | 3 | 4 | 5 |
| 11. The arrangement of the intervention fits my schedule. | 1 | 2 | 3 | 4 | 5 |
| 12. The intervention booklet contains comprehensive knowledge and is easy to understand. | 1 | 2 | 3 | 4 | 5 |
| **Intervention Effects** | | | | | |
| 13. The intervention enhanced my understanding of colorectal cancer and cancer-related fatigue. | 1 | 2 | 3 | 4 | 5 |
| 14. The intervention guided me on engaging in healthy physical activity, dietary behaviours, and sleep hygiene. | 1 | 2 | 3 | 4 | 5 |
| 15. The intervention assisted me in coping with negative thoughts and emotions. | 1 | 2 | 3 | 4 | 5 |
| **16. Overall, I am satisfied with this intervention.** | 1 | 2 | 3 | 4 | 5 |

**Supplementary Table S5.** Semi-structured interview outline

**1. Reflecting on Experiences in the Intervention Sessions**

- Can you describe any experiences that were especially impactful for you?
- Were there any aspects of the intervention that you found particularly positive or negative? What factors contributed to these feelings?
- How do you perceive the format of the intervention? Do you have any suggestions for improvement?
- What are your thoughts on the delivery mode of the intervention? Are there any changes you would recommend?
- How do you evaluate the overall structure and arrangement of the sessions? Which elements did you find appropriate and well-organised? Do you have any suggestions for enhancement?

**2. Exploring Perceived Benefits and Facilitators of the Intervention**

- Which aspects of the intervention do you consider to be the most beneficial? Can you elaborate on your reasoning?
- Have you observed any changes in your management of cancer-related fatigue following your participation in the intervention sessions?
- Have there been any noticeable changes in your physical activity, dietary behaviours, or sleep hygiene as a result of the intervention?
- How has your approach to coping with negative emotions evolved since participating in the intervention?
- Which facilitators do you believe positively contributed to the effectiveness of the intervention? Can you provide any specific reasons or details?

**3. Identifying Areas for Improvement and Barriers of the Intervention**

- Are there any components of the intervention that you found unhelpful or less effective? Could you explain why?
- What elements of the structure and sessions do you perceive as inappropriate?
- Which aspects of the intervention did you find difficult to understand? Can you elaborate on specific reasons or details?
- Are there any components that you struggled to adopt? Please provide specific examples or explanations.
- What barriers do you face in participating in the intervention session? Can you provide any specific reasons or details?

**4. Additional Feedback and Suggestions**

- Do you have any further feedback or suggestions regarding the intervention?

**Qualitative data analysis methods:** Qualitative data from the semi-structured interviews were analysed using conventional content analysis, an inductive approach that derives coding categories directly from the data without imposing preconceived theoretical frameworks [1]. All interviews were audio-recorded, transcribed verbatim, and organised in NVivo version 15. The analysis followed four sequential steps: 1) familiarising with the data through repeated reading of the transcripts; 2) initial coding by identifying and extracting meaningful units directly from the data without imposing preconceived categories; 3) organising codes into categories and sub-categories by comparing their similarities and differences; and 4) refining categories and sub-categories through iterative review. Theoretical saturation was used as the criterion for determining analytical sufficiency, with sufficiency considered achieved when no new categories or sub-categories emerged from additional interviews, which occurred after eight interviews. To ensure methodological rigour, the principal investigator (LJL) and a research assistant, who are both bilingual in English and Mandarin and experienced in qualitative data analysis, independently conducted the coding and analysis, and resolved disagreements through discussion with a third senior researcher (YYC) to reach consensus. All analyses were conducted in Chinese to preserve linguistic nuances. The principal investigator translated the categories, sub-categories, and representative examples into English, and the bilingual research assistant verified semantic equivalence.

**Supplementary Table S6.** Outcome measurements for primary and secondary outcomes

| **Outcomes** | **Instruments** |
| --- | --- |
| Socio-demographic and clinical characteristics | Socio-demographic measures consisted of age, sex, marital status, educational level, employment status, monthly income, medical insurance, place of residence, and living arrangements. Clinical measures included body mass index, diagnosis, cancer stage, time since treatment completion, treatments have received, stoma status, number of comorbidities, haemoglobin, and albumin. |
| Cancer-related fatigue | The Cancer Fatigue Scale (CFS) is a 15-item self-report instrument measuring three dimensions of CRF: physical, cognitive, and emotional fatigue [2]. Each item is rated on a 5-point Likert scale, ranging from 1 (no) to 5 (very much). The total scores range from 0 to 60, with scores ≥ 18 indicating clinically meaningful CRF [3]. The Chinese version has demonstrated acceptable internal consistency, with Cronbach's alpha coefficients of 0.85, 0.81, 0.63, and 0.86 for physical, cognitive, emotional, and total fatigue, respectively; and established test-retest reliability, with Intraclass Correlation Coefficients (ICCs) of 0.75, 0.55, 0.77, and 0.65 [4]. |
| Anxiety symptoms | The Generalised Anxiety Disorder Questionnaire-7 (GAD-7) 7-item self-report scale measuring anxiety symptoms over the past two weeks [5]. Items are scored on a 4-point Likert scale, ranging from 0 (not at all) to 3 (nearly every day). The total scores range from 0 to 21, with scores of 0-4, 5-9, 10-14, and 15-21 indicating minimal, mild, moderate, and severe anxiety, respectively. The Chinese version has demonstrated good internal consistency in cancer patients, with a Cronbach's alpha coefficient of 0.91 [6]. |
| Depressive symptoms | The Patient Health Questionnaire-9 (PHQ-9) is a 9-item self-report scale measuring depressive symptoms over the past two weeks [7]. Items are rated on a 4-point Likert scale, ranging from 0 (not at all) to 3 (nearly every day). The total scores range from 0 to 27, with scores of 0-4, 5-9, 10-14, and 15-19, 20-27 representing minimal, mild, moderate, moderately severe, and severe depressive symptoms, respectively. The Chinese version has demonstrated good internal consistency, with a Cronbach's alpha coefficient of 0.91 [6]. |
| Physical activity | The physical activity domain of the Health-Promoting Lifestyle Profile-Ⅱ (HPLP-Ⅱ) measures physical activity through eight self-report items [8]. Items are rated on a 4-point Likert scale, ranging from 1 (never) to 4 (routinely). The total scores range from 8 to 32, with higher scores indicating higher level of physical activity. The Chinese version has indicated good internal consistency, with a Cronbach's alpha coefficient of 0.83 [8]. |
| Dietary behaviours | The nutrition domain of the HPLP-Ⅱ measures dietary behaviours through six self-report items [8]. Items are rated on a 4-point Likert scale, ranging from 1 (never) to 4 (routinely). The total scores range from 9 to 36, with higher scores indicating healthier dietary behaviours. The Chinese version has demonstrated good internal consistency, with a Cronbach's alpha coefficient of 0.71 [8]. |
| Sleep disturbance | The Patient-Reported Outcomes Measurement Information System Sleep Disturbance-Short Form 4a (PROMIS SD-SF-4a) consists of four self-report items measuring sleep disturbance over the past week [9]. Items are rated on a 5-point Likert scale, ranging from 1 to 5. Raw scores are converted to standardized T-scores according to the PROMIS scoring manual. The total T-scores range from 32.0 to 73.3, with higher scores indicating greater sleep disturbance. The Chinese version has demonstrated acceptable internal consistency among cancer patients, with a Cronbach's alpha coefficient of 0.79 [10]. |

**Supplementary Table S7.** Perceptions of colorectal cancer survivors for participating in the psycho-behavioural intervention

The main categories, sub-categories, and supporting verbatim examples are summarised below, and more details are presented in the subsequent table.

***Addressing Unmet Support and Information Needs after Treatments***

**Providing Post-Treatment Professional Support.** All participants (n = 11) demonstrated a lack of professional post-treat support, while the intervention filled in this gap by providing weekly, structured sessions. For example, S7 articulated this: “*After my discharge from the hospital, I have few opportunities to receive professional support. This programme helps me a lot, and I eagerly anticipate our weekly communications because each session provides me with new skills for my recovery, as well as the opportunity to ask questions and discuss any challenges I encounter.*” S3 similarly reflected: “*I have never met a programme like this, and feel lucky to participate in the study.*”

**New Understanding of Cancer-Related Fatigue and Symptom Management.** Nearly all participants (n = 10) received critical knowledge about CRF, which had caused their confusion. For example, S1 stated: “*This is the first time I have heard about cancer-related fatigue. I originally thought my fatigue was just due to lack of rest. I kept wondering why I still so exhausted, even though I was sleeping plenty every day.*” Beyond CRF, the intervention equipped participants with concrete guidance on managing post-treatment symptoms. S6 noted: “*Before, when I experience symptoms like diarrhoea or constipate, I just felt helpless. Now, I can refer to the booklet for answers on what to do.*”

***Developing Practical Skills for Physical and Emotional Challenges***

**Translating Health Goals into Achievable Behavioural Steps.** The majority of participants (n = 9) reported that rather than merely receiving passive support, they were also equipped with practical skills to engage in healthy behaviours by deconstructing complex wellness goals into manageable, actionable steps. For example, S2 shared: “*I always knew that eating right, staying active, and sleeping well were crucial for my recovery, but I just couldn’t put them into practice. These sessions helped me break these seemingly daunting tasks into smaller, manageable steps, gradually helping me start them.*” S8 shared a similar experience: “*After our discussions, I find the steps to follow through and exercise. This allows me to update you on my progress during our conversations each week.*”

**Developing Emotional Regulation Techniques for Real-Life Stressors.** For emotional challenges, nine participants reported that the intervention provided techniques to regulate negative emotions and thoughts in real-life situations. For example, S1 reflected: “*I believe the most beneficial aspect of the course is the section focused on emotion expression. I had few opportunities to exploring my thoughts and emotions in this way before. I genuinely feel more relaxed during each session, and I am getting better at dealing with what is going on inside my head.*” Critically, participants applied these techniques to address stressors in their real-life. S2 provided a concrete example: “*As you know, I have to conduct regularly follow-up appointments every three months, and my relationship with my mother-in-law is quite strained. These things used to send me spiraling — I would overthink everything and my emotions would be all over the place. But now I can catch myself when those negative thoughts start up and use the emotion expression techniques to work through them.*”

***Suggestions for Improving Intervention Delivery***

No major implementation difficulties were reported by participants, however, participants identified three areas for refinement that could enhance intervention delivery and accessibility.

**Digital Format for the Information Booklet.** One participant who had returned to work found that the hard copy is difficult to carry daily. For example, S5 stated: “*I need to work every day, making it impractical to carry a hard copy of the information booklet with me. I think proving a soft copy would be more convenient.*”

**Online Option for the First Face-to-Face Session.** Four participants reported that the mandatory face-to-face first session created scheduling conflicts with medical appointments and travel. For example, S5 described: “*Every time I visit the outpatient clinic for a follow-up examination, I have to undergo numerous medical tests, each with its own time constraints. Therefore, during our first face-to-face session, I felt a bit worried that I might miss these tests.*” S6 added: “*I felt a bit rushed during our first session because I had to catch my train back home, which is in another city. It would have been much better if the first session could have been online too, just like all the other ones.*”

**Earlier Session Reminders for Schedule Planning.** One participant suggested to send reminders earlier, allowing them to plan their own activities in advance. For example, S3 suggested: “*You reminded me one day in advance before each session. However, it would be even better if you could remind me earlier, allowing me to plan my schedule more effectively.*”

| Theme | Sub-theme | Examples |
| --- | --- | --- |
| **Addressing unmet support and information needs after treatments** | Providing post-treatment professional support | S3: I have never met a programme like this, and feel lucky to participate in the study. This program has definitely guided my recovery after completing treatment by addressing multiple dimensions, including providing health information, helping me lead a healthier lifestyle, and assisting me in managing negative thoughts and emotions.  S7: After my discharge from the hospital, I have few opportunities to receive professional support. This programme helps me a lot, and I eagerly anticipate our weekly communications because each session provides me with new skills for my recovery, as well as the opportunity to ask questions and discuss any challenges I encounter. |
|  | New understanding of CRF and symptom management | S1: This is the first time I have heard about cancer-related fatigue. I originally thought my fatigue was just due to lack of rest. I kept wondering why I still so exhausted, even though I was sleeping plenty every day.  S2: The information booklet includes instructions on how to handle common side effects that may arise after treatment, which is huge for me. This section is particularly important because we deal with these issues all the time but usually have no idea how to fix them.  S6: Before, when I experience symptoms like diarrhoea or constipate, I just felt helpless. Now, I can refer to the booklet for answers on what to do. |
| **Developing practical skills for physical and emotional challenges** | Translating health goals into achievable behavioural steps | S2: I always knew that eating right, staying active, and sleeping well were crucial for my recovery, but I just couldn’t put them into practice. These sessions helped me break these seemingly daunting tasks into smaller, manageable steps, gradually helping me actually start doing them.  S4: When I don't feel like moving, I force myself to complete the homework we discussed and assigned last week. It sounds backward, but I actually feel less tired afterwards.  S8: Sometimes I am too lazy to engage in physical activity, but after our discussions, I find the steps to follow through and exercise. This allows me to update you on my progress during our conversations each week. |
|  | Developing emotional regulation techniques for real-life stressors | S1: I believe the most beneficial aspect of the course is the section focused on emotion expression. I had few opportunities to exploring my thoughts and emotions in this way before. I genuinely feel more relaxed during each session, and I am getting better at dealing with what is going on inside my head.  S2: As you know, I have to conduct regularly follow-up appointments every three months, and my relationship with my mother-in-law is quite strained. These things used to send me spiralling — I would overthink everything and my emotions would be all over the place. But now I can catch myself when those negative thoughts start up and use the emotion expression techniques to work through them.  S7: When I get overwhelmed by distress, I practice the emotion expression techniques we learned, and usually I feel much better and more settled. |
| **Suggestions for improving intervention delivery** | Digital format for the information booklet | S5: I need to work every day, making it impractical to carry a hard copy of the information booklet with me. I think proving a soft copy would be more convenient. |
|  | Online option for the first face-to-face session | S5: Every time I visit the outpatient clinic for a follow-up examination, I have to undergo numerous medical tests, each with its own time constraints. Therefore, during our first face-to-face session, I felt a bit worried that I might miss these tests.  S6: I felt a bit rushed during our first session because I had to catch my train back home, which is in another city. It would have been much better if the first session could have been online too, just like all the other ones. |
|  | Earlier session reminders for schedule planning | S3: You commonly remind me one day in advance before each session. However, it would be even better if you could remind me earlier, allowing me to plan my schedule more effectively. |

**References:**

1. Hsieh, H. F., & Shannon, S. E. Three approaches to qualitative content analysis. Qualitative Health Research 2005; 15(9), 1277-1288.

2. Okuyama T, Akechi T, Kugaya A, Okamura H, Shima Y, Maruguchi M, Hosaka T, Uchitomi Y. Development and validation of the cancer fatigue scale: A brief, three-dimensional, self-rating scale for assessment of fatigue in cancer patients. Journal of Pain and Symptom Management 2000; 19(1):5-14.

3. Okuyama T, Tanaka K, Akechi T, Kugaya A, Okamura H, Nishiwaki Y, Hosaka T, Uchitomi Y. Fatigue in ambulatory patients with advanced lung cancer: Prevalence, correlated factors, and screening. Journal of Pain and Symptom Management 2001; 22(1):554-564.

4. Zhang F, Ding Y, Han L. Reliability and validity of the Chinese version of Cancer Fatigue Scale. Chinese Mental Health Journal 2011; 25(11):810-813.

5. Spitzer RL, Kroenke K, Williams JB, Löwe B. A brief measure for assessing generalized anxiety disorder: the GAD-7. Archives of internal medicine 2006; 166(10):1092-1097.

6. Chen X, Wang L, Liu L, Jiang M, Wang W, Zhou X, Shao J. Factors associated with psychological distress among patients with breast cancer during the COVID-19 pandemic: a cross-sectional study in Wuhan, China. Supportive Care in Cancer 2021; 29:4773-4782.

7. Kroenke K, Spitzer RL, Williams JB. The PHQ‐9: validity of a brief depression severity measure. Journal of general internal medicine 2001; 16(9):606-613.

8. Cao W, Guo Y, Ping W, Zheng J. Development and psychometric tests of a Chinese version of the HPLP-Ⅱ Scales. Chinese Journal of Disease Control & Prevention 2016; 20(3):286.

9. Cella D, Choi SW, Condon DM, Schalet B, Hays RD, Rothrock NE, Yount S, Cook KF, Gershon RC, Amtmann D et al. PROMIS® Adult Health Profiles: Efficient Short-Form Measures of Seven Health Domains. Value in Health 2019; 22(5):537-544.

10. Zhang Q, Zhao J, Liu Y, Cui Y, Wang W, Li J, Liu Y, Tian F, Wang Z, Zhang H et al. Evaluating the psychometric properties of the simplified Chinese version of PROMIS-29 version 2.1 in patients with hematologic malignancies. Sci Rep 2024; 14(1):11153.
